# Comparison of the performance of SAG2, GRA6, and GRA7 for serological diagnosis of *Toxoplasma gondii* infection in cats

**DOI:** 10.3389/fvets.2024.1423581

**Published:** 2024-06-05

**Authors:** Serges Sabukunze, Haorong Gu, Lin Zhao, Honglin Jia, Huanping Guo

**Affiliations:** ^1^State Key Laboratory for Animal Disease Control and Prevention, Harbin Veterinary Research Institute, Chinese Academy of Agricultural Sciences, Harbin, China; ^2^Heilongjiang Research Center for Veterinary Biopharmaceutical Technology, Harbin Veterinary Research Institute, Chinese Academy of Agricultural Sciences, Harbin, China

**Keywords:** *Toxoplasma gondii*, SAG2, GRA3, GRA6, GRA7, serodiagnosis, cats

## Abstract

Toxoplasmosis is an important zoonotic disease caused by *Toxoplasma gondii* that can infect almost all warm-blooded animals worldwide, including humans. The high prevalence of *T. gondii* infection and its ability to cause serious harm to humans and animals, especially immunodeficient individuals, make it a key public health issue. Accurate diagnostic tools with high sensitivity are needed for controlling *T. gondii* infection. In the current study, we compared the performance of recombinant SAG2, GRA6, and GRA7 in ELISA for the serological diagnosis of *T. gondii* infection in cats. We further investigated the antigenicity of recombinant dense granule protein 3 (rGRA3), rGRA5, rGRA8, and rSRS29A expressed in a plant-based, cell-free expression system for detecting antibodies in *T. gondii*-infected cats. In summary, our data suggest that GRA7 is more sensitive than the other two antigens for the serodiagnosis of *T. gondii* infection in cats, and GRA3 expressed in the cell-free system is also a priming antigen in serological tests for detecting *T. gondii* infection in cats.

## 1 Introduction

*Toxoplasma gondii*, an obligate protozoan parasite, is responsible for toxoplasmosis. *T. gondii* infection is ranked as the fourth most common food-borne parasitic infection globally by the World Health Organization and the Food and Agriculture Organization of the United Nations (1). It is estimated that more than one-third of the world’s population has been infected by *T. gondii* (2). The high prevalence of *T. gondii* infection has become a public health issue, and the parasite is seriously harmful to the global population, especially for pregnant women and immunocompromised individuals (3, 4). *T. gondii* infection also results in large economic losses in the livestock sector because of the wide host area. Felines such as cats, as the final host of *T. gondii*, play an important role in the transmission of toxoplasmosis to humans and animals by excreting oocysts and contaminating the environment. The increasing population of domestic cats in close contact with humans is becoming a key source of *T. gondii* infection in humans (5, 6). Furthermore, an incalculable number of stray cats increase the prevalence of toxoplasmosis and related public health problems. The development of reliable diagnostic methods for determining feline *T. gondii* infection is vital to prevent and control the transmission of *T. gondii* in humans and animals.

Enzyme-linked immunosorbent assay (ELISA), indirect hemagglutination assay, and Western blotting are often used to detect antibodies against *T. gondii* (7). Among them, ELISA is the most suitable for epidemiological surveillance of a large number of samples. Whole parasite or parasite lysate-based tests are generally considered the gold standard for serological diagnosis. Surface antigens (SAGs) are the most abundant proteins on the parasite’s surface and are involved in the processes of adhesion and invasion. Dense granule proteins (GRAs), as the major components of excretory-secretory antigens (ESAs) expressed by *T. gondii* (8, 9), are released into the intravacuolar network (IVN), inserted in the PVM, and delivered into the lumen of the PV or host cells (10) after invasion. Several SAGs or GRAs have been expressed as recombinant proteins and used in serological tests to detect IgG or IgM in serum samples (11). However, the usefulness of these antigens in the tests still needs to be evaluated further. In a previous study, the performance of recombinant GRA6 and GRA7 in serological tests was compared for detecting human *T. gondii* infection (12). Recombinant SAG1 and GRA7 have also been utilized for serodiagnosis for *T. gondii* infection in cats (13). GRA3 is a type I transmembrane protein. Previous studies have shown that GRA3 has the potential to be used as an antigen (14, 15), but the usefulness of the full length of this protein has not been evaluated in serological tests.

In this study, we carried out a comparative analysis of the performance of recombinant SAG2, GRA6, and GRA7 for the serodiagnosis of *T. gondii* infection in cats. Additionally, we explored the potential of a recombinant GRA3 as an alternative antigen.

## 2 Materials and methods

### 2.1 Serum samples

A total of 73 serum samples from cats, collected from Qinghai Province, China, were used in our study to compare the performance of the recombinant proteins in the ELISAs (13). These samples, along with a serum against *Toxoplasma* collected from a cat experimentally infected with the *Toxoplasma* RH strain and a negative serum collected from a non-infected cat and kept in our lab, were used to optimize the conditions of the ELISAs in this study. A serum from a goat experimentally infected with the *Toxoplasma* Me49 strain and the pre-immunized serum (a gift from Dr. Bang Shen) were used to test the reactivity of the recombinant proteins with positive serum against the Me49 strain parasites.

### 2.2 Expression and purification of the recombinant SAG2, GRA6, and GRA7

First, specific PCR primers were designed to amplify the gene products of SAG2, GRA6, and GRA7 from the cDNA of RH strain parasites. The PCR products of GRA6 were inserted into the pET-32a expression vector by *EcoR* I using a ClonExpress II One-Step cloning kit (C112-02, Vazyme, China) according to the manufacturer’s protocol. The PCR products of SAG2 and GRA7 were cloned into the pColdII vector. The recombinant plasmids were transformed into competent *E. coli* BL21 (DE3) (EC1002, Vazyme, China) cells to express the recombinant proteins. The resulting recombinant GRA6 (rGRA6) was fused with a TrxA and an S tag, except for the 6 × His tag. The resulting recombinant SAG2 (rSAG2) and GRA7 (rGRA7) were fused with a 6 × His tag. The recombinant proteins were purified according to standard methodology. In brief, the bacterial culture was centrifuged at 8, 000 rpm for 10 min. The cell pellets were collected and suspended in 25 mL of pre-chilled lysis buffer, followed by sonication using ultrasound (VCX-750/130 ultrasonic crusher, SONICS, United States) to disturb the cell pellet. After sonication, the suspension was centrifuged for 10 min at 13,000 rpm at 4°C, and the supernatant was collected. Then, 1 mL of Ni-NTA Superflow resin (L00250-100, GenScript, United States) was washed five times with lysis buffer and mixed with the supernatants, and the samples were rotated overnight at 4°C. Then, the rSAG2, rGRA6, and rGRA7 were eluted with 1 mL of elution buffer.

### 2.3 Evaluation of ELISAs based on the rSAG2, rGRA6, and rGRA7 for detecting antibodies in cat sera

Indirect ELISAs based on the recombinant proteins were established and optimized. The purified rSAG2 (0.5 μg), rGRA6 (1 μg), and rGRA7 (0.5 μg) diluted in carbonate buffer (pH 9.6) were coated on the plates and incubated overnight at 4°C. The plates were washed once with PBST (0.05% Tween in PBS, v/v), blocked with 5% skimmed milk in PBS, and incubated for 2 h at 37°C. After washing the plates as above, serum samples were diluted at 1:100 and added to the wells. The plates were incubated for 1 h at 37°C and washed six times. Thereafter, horseradish peroxidase (HRP)-conjugated anti-cat IgG diluted at 1:5000 was added and incubated for 1 h at 37°C. Following extensive washing five times with PBST, the color was developed by adding 100 μL/well tetramethylbenzidine substrate solution (PA107, TIANGEN, China), stopped with 2 M (7664-93-9, Sigma, United States), and reactivity was determined using the ELISA microplate reader at OD_450nm_ absorbance.

An ELISA based on *T. gondii* lysate (TgLys-ELISA) was used as a reference method (16) to compare the efficiency of rSAG2, rGRA6, and rGRA7 for detecting antibodies in cat sera. The cutoff values of the ELISAs, including the TgLys-ELISA, were calculated with the mean OD value and 3 × SD of 30 negative samples, which were confirmed as negative using an agglutination test (17, 18). Totally, 73 cat sera were examined by the ELISAs, and each sample was detected in duplicate in the ELISAs.

### 2.4 Expression of recombinant proteins using a plant-based cell-free system

The recombinant proteins were expressed using a cell-free protein expression kit (WEPRO 7240, CellFree Sciences Co., Japan) according to the manufacturer’s protocol. In brief, the gene fragments were amplified using gene-specific primers (Supplementary Table S1) from the cDNA of *T. gondii* RH tachyzoites combined with the primer HA-MCS-R: 5’-ACGTCGCGACCGCGGACAAGATCTTCAagcgtaatccggcac-3′ by overlapping PCR. The amplified gene sequences were fused with an HA-tag sequence. Then, the purified PCR fragments with the HA-tag were inserted into a pEU-E01-MCS vector. The purified plasmids were used for mRNA transcription and protein translation using the expression kit. The expression of the recombinant proteins was examined using the Western blot analysis.

### 2.5 Evaluation of the antigenicity of the recombinant proteins expressed in the cell-free system

The antigenicity of the recombinant proteins was examined through an ELISA method. Briefly, a plate was coated with 1 μL of anti-HA antibody (51064-2-AP, Proteintech, China) (1:50 μL of carbonate buffer) per well and was incubated at 4°C overnight. The plate was washed once with PBST (0.05% Tween in PBS, v/v), blocked for 2 h at 37°C, and then washed with PBST five times. The translated solution containing the recombinant proteins was diluted in an equal volume of skim milk and added into the wells coated with the anti-HA antibodies. The plate was then incubated for 2 h. The remaining procedure is similar to the above ELISAs except that the serum samples were incubated for 2 h, and the second antibodies were diluted at 1:1500 and incubated for 2 h.

### 2.6 Data analysis

The ELISA results were processed using Microsoft Excel. The sensitivity and specificity of the ELISAs were calculated as follows: sensitivity = number of true positives/(number of true positives + number of false negatives) and specificity = number of true negatives/(number of true negatives + number of false positives). The kappa values were calculated using an online statistical tool (http://vassarstats.net/ accessed on 9 June 2021). Graphs were produced using GraphPad Prism version 8.0.2 (GraphPad Software Inc. La Jolla, CA, United States).

## 3 Results

### 3.1 Expression and purification of rSAG2, rGRA6, and rGRA7 expressed in bacteria

**Figure 1:**
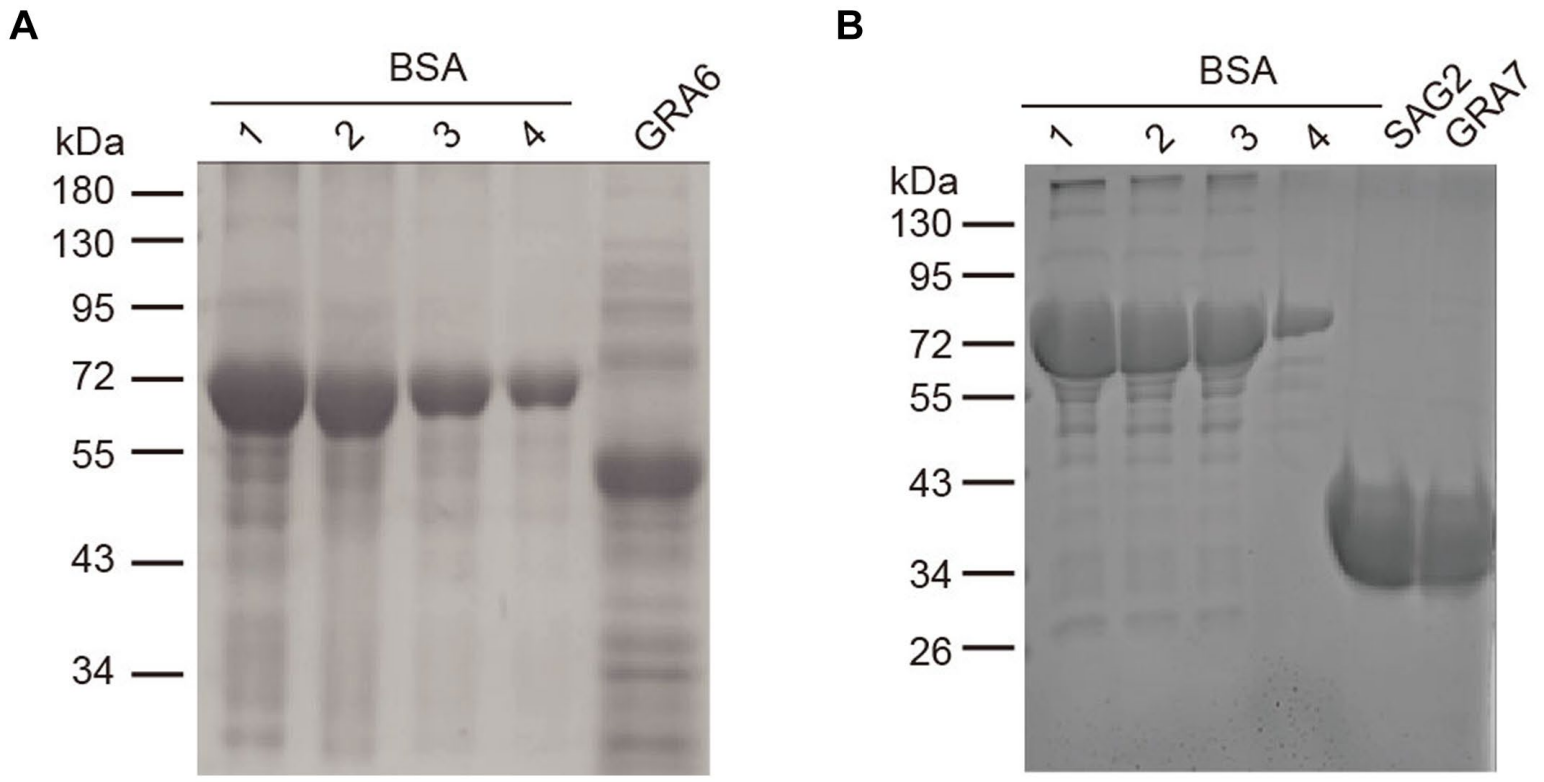
SDS-PAGE analysis of the purified rSAG2, rGRA6, and rGRA7. **(A)** Purified rGRA6 fused with His. **(B)** Purified rSAG2 and rGRA7 fused with His. A BSA standard solution was diluted into the concentrations as indicated in the figure and used to quantify the concentration of the recombinant proteins. 1, 2000 ng/μL; 2, 1,500 ng/μL; 3, 1,000 ng/μL; and 4, 500 ng/μL.

rSAG2, rGRA6, and rGRA7 were expressed in *E. coli* as His tag-fused proteins. The results showed that all of the three proteins could be effectively expressed and purified from the *E. coli* lysates. As shown in Figure 1, the sizes of the purified rSAG2 (30 kDa), rGRA6 (50 kDa), and rGRA7 (30 kDa) were bigger than their predicted sizes but similar to the results in previous studies (19, 20).

### 3.2 Evaluation of ELISAs based on rSAG2, rGRA6, and rGRA7 to detect *Toxoplasma* infection in cats

In total, 73 cat serum samples were examined using ELISAs based on rSAG2, rGRA6, and rGRA7. The rSAG2-ELISA identified 35 positive samples (47.95%) and 38 negative samples (52.05%). The rGRA6-ELISA identified 36 positive samples (49.32%) and 37 negative samples (50.68%), while the rGRA7-ELISA identified 39 positive samples (53.42%) and 34 negative samples (46.58%) (Table 1). The rSAG2-ELISA showed the lowest sensitivity, and the rGRA6-ELISA showed a lower sensitivity (90%) than the TgLys-ELISA, although with 100% specificity. The rGRA7-ELISA had the highest sensitivity (97.5%), specificity (100%), and kappa values (0.97) (Table 2).

**Table 1:** Comparison of the performance of rSAG2, rGRA6, and rGRA7 in ELISAs for detecting antibodies in cats (*N* = 73).

TgLys	rSAG2 (%)		rGRA6 (%)		rGAR7 (%)	
	(−)	(+)	(−)	(+)	(−)	(+)
Negative (−)	33 (45.21)	0	33 (45.21)	0	33 (45.21)	0
Positive (+)	5 (6.84)	35 (47.95)	4 (5.47)	36 (49.32)	1 (1.37)	39 (53.42)
**Total**	**38** (52.05)	**35** (47.95)	**37** (50.68)	**36** (49.32)	**34** (46.58)	**39** (53.42)

TgLys, *Toxoplasma gondii* lysate.

**Table 2:** Sensitivity and specificity of different ELISAs based on rSAG2, rGRA6, and rGRA7.

Entitled	rSAG2	rGRA6	rGRA7
Sensitivity (%)	87.5	90	97.5
Specificity (%)	100	100	100
Kappa value	0.86	0.89	0.97

### 3.3 Expression of recombinant proteins in the cell-free system

**Figure 2:**
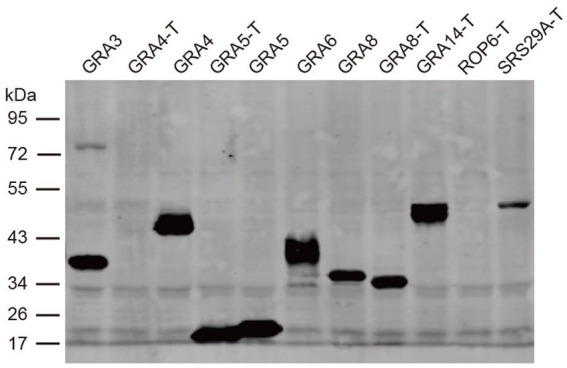
Western blot analysis of the expression of recombinant proteins in the cell-free system. The expression of the recombinant proteins was detected with an anti-HA antibody. T is the protein without transmembrane domains.

The above results indicated that none of the three proteins is sufficient for detecting antibodies in cats. Therefore, we tried to express more antigens, including GRA3, GRA4, GRA5, GRA8, GRA14, ROP6, and SRS29A, to investigate whether they could be used as alternative antigen antigens for serodiagnosis. These antigens showed good potential for the detection of antibodies in a previous study (14). However, most of these proteins could not be efficiently expressed in *E. coli* (data not shown). We then tried to express these antigens and/or their truncated versions in a wheat germ cell-free system. Western blotting using an anti-HA-flag antibody showed that all of the antigens were successfully expressed except the truncated version of GRA4 and ROP6 (Figure 2).

### 3.4 Evaluation of the antigenicity of the recombinant proteins expressed in the cell-free system

**Figure 3:**
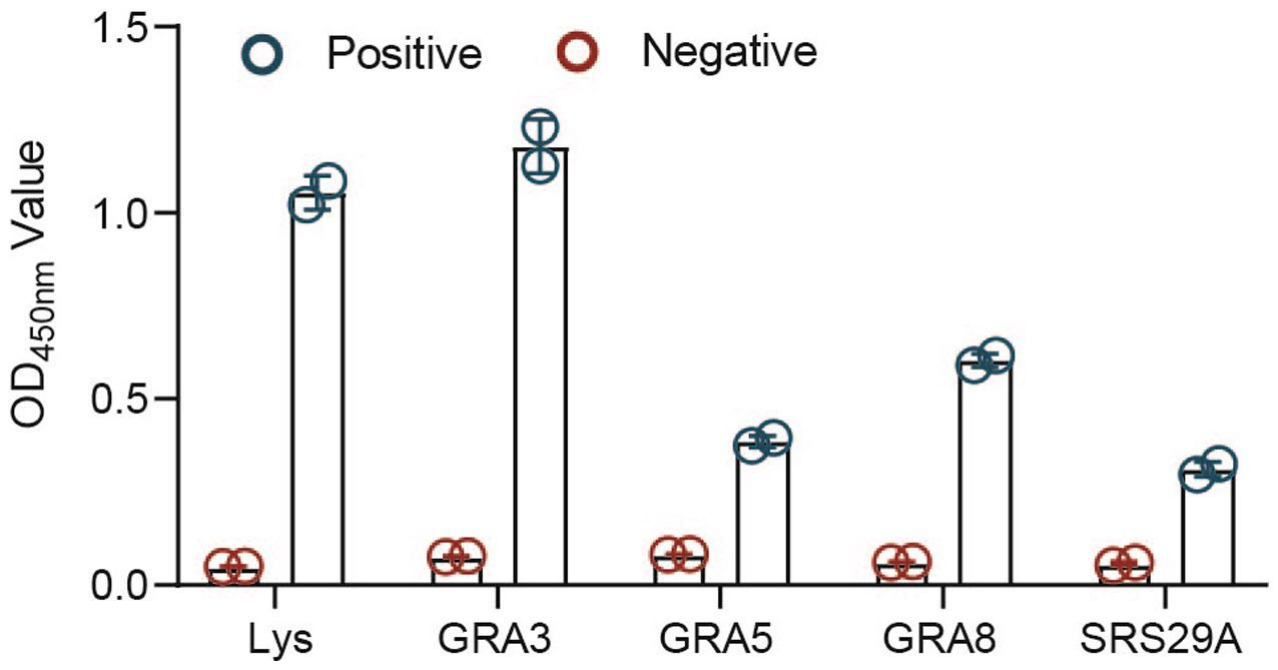
Antigenicity analysis of the selected recombinant proteins expressed in the cell-free system. Antigenicity analysis of the selected proteins expressed in the cell-free system was detected through an ELISA. TgLys was coated as a control.

The proteins were then used to examine a positive serum sample in an ELISA to test their potential in diagnostic tests. A positive serum from an animal experimentally infected with an ME49 strain was used in this experiment instead of the cat serum. Using the serum from an animal infected with a different parasite strain might help increase the possibility of finding a more sensitive antigen. We found that all of the selected recombinant proteins (GRA3, GRA5, GRA8, and SRS29A) could react with the positive serum but not with the negative control. Among them, the recombinant GRA3 (*CF*-rGRA3) seems to react with the positive serum more efficiently (Figure 3).

**Figure 4:**
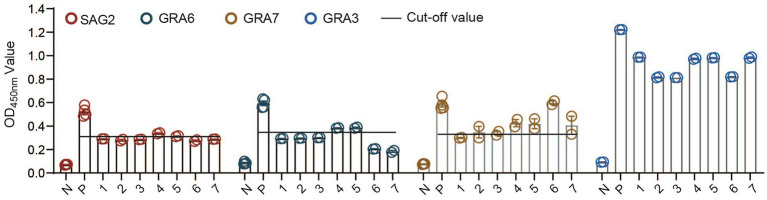
Antigenicity analysis of the *CF*-rGRA3 using cat serum samples. TgLys, *T. gondii* lysate; P: a serum from a cat experimentally infected with *T. gondii*; PRS: a random positive sample in TgLys-ELISA; 1, 2, 3, 4, 5, 6, and 7 represent the selected cat samples.

Therefore, we decided to test whether the *CF*-rGRA3 could differentiate the TgLys-ELISA-positive samples that showed weak reactions or were negative in the ELISAs based on rSAG2, rGRA6, or rGRA7 from the negative control. Seven cat sera were used to examine the potential of *CF*-rGRA3 for the examination. The results showed that *CF*-rGRA3 strongly reacted with antibodies in these samples but not in the negative control (Figure 4).

## 4 Discussion

Felines are only the definitive host of *T. gondii*, and the relationship between cats as companion animals and humans increases the risk of *T. gondii* infection for humans (21). Screening infected cats using reliable diagnostic methods is one of the most important ways to control the transmission of toxoplasmosis. Serological tests based on a prepared extract of whole *T. gondii* tachyzoites have advantages such as high sensitivity and specificity, but the preparation of parasite lysate is very time-consuming, poses an infection risk, and necessitates inconvenient parasite culture. Due to these reasons, intensive research is being carried out on the usefulness of recombinant antigens to replace TgLys in the tests (22, 23).

In this study, three antigens were expressed as His tag-fused proteins to compare their performance in serological tests. We cannot exclude the possibility of false positive results caused by the tags fused with the recombinant proteins in the ELISAs. However, compared with the result of the TgLys-ELISA, there are few false positive samples in the ELISAs based on the recombinant proteins. Therefore, we think that it is acceptable to use tag-fused proteins in the ELISAs. The rGRA6-ELISA developed in the current study exhibited low sensitivity to detect IgG antibodies in cat sera. In a previous study, a rGRA6-ELISA had a 96% sensitivity for detecting IgG (24). Another ELISA, using rGRA6 with a His tag, detected recent *T. gondii* infection with higher sensitivity (93.9%) than chronic infection (60.6%) (12, 19). To the best of our knowledge, our study is the first to investigate rGRA6 as an ELISA antigen for detecting toxoplasma infection in cats. GRA7 has been previously validated as a serodiagnosis antigen for toxoplasma infection in cats from several different countries (25–27). Moreover, GRA7 is a good ELISA-based serodiagnosis marker for human toxoplasmosis, with a specificity of 98–100% and sensitivity of 81–98.9% (12, 28, 29). Although the specificity and sensitivity of the rGRA7-ELISA were comparable with those of the TgLys-ELSIA in this study, neither this antigen nor rGRA6 was sensitive enough to detect *T. gondii* infection in cats. In this study, the *CF*-rGRA3-ELISA showed high antigenicity. These preliminary results suggest that *CF*-rGRA3 might be an alternative antigen for the serodiagnosis of *T. gondii* infection.

GRAs are well-recognized among *T. gondii* antigens for their excellent diagnostic ability (30). However, these antigens were sometimes difficult to express in *E. coli* in our previous experience. In the current study, we successfully expressed *T. gondii* proteins using a plant cell-free system. The results revealed that IgG antibodies could be detected efficiently using the selected recombinant proteins expressed in the system. Our data suggest that the plant expression system could be an efficient way to characterize antigens used for serological tests. However, the application of cell-free systems is still limited because of their high cost. Therefore, we did not determine the sensitivity and specificity of the *CF*-rGRA3 with a large number of serum samples, but rather examined the potential of this antigen to differentiate the weak positive samples from the negative ones in this study.

Recently, the methodology has become more productive and applicable for large-scale protein production due to the utilization of less-expensive energy resources (31). Future investigation and improvements will help overcome these limitations and extend the potential of the cell-free protein synthesis method to express antigens for serological tests (32).

## Data availability statement

The raw data supporting the conclusions of this article will be made available by the authors, without undue reservation.

## Ethics statement

Ethical approval was not required for the studies on animals in accordance with the local legislation and institutional requirements because only commercially available established cell lines were used.

## Author contributions

SS: Investigation, Formal analysis, Writing – original draft. HaG: Writing – review & editing, Investigation. LZ: Writing – review & editing, Investigation. HJ: Validation, Resources, Project administration, Methodology, Funding acquisition, Conceptualization, Writing – review & editing, Writing – original draft. HuG: Writing – review & editing, Writing – original draft, Supervision, Formal analysis.

## References

[1] Torgerson PR Devleesschauwer B Praet N Speybroeck N Willingham AL Kasuga F. World Health Organization estimates of the global and regional disease burden of 11 foodborne parasitic diseases, 2010: a data synthesis. PLoS Med. (2015) 12:e1001920. doi: 10.1371/journal.pmed.1001920, PMID: 26633705 PMC4668834

[2] Rahmanian V Rahmanian K Jahromi AS Bokaie S. Seroprevalence of toxoplasma gondii infection: An umbrella review of updated systematic reviews and meta-analyses. J Family Med Prim Care. (2020) 9:3848–55. doi: 10.4103/jfmpc.jfmpc_753_20, PMID: 33110778 PMC7586519

[3] Shirbazou S Delpisheh A Mokhetari R Tavakoli G. Serologic detection of anti toxoplasma gondii infection in diabetic patients. Iran Red Crescent Med J. (2013) 15:701–3. doi: 10.5812/ircmj.5303, PMID: 24578838 PMC3918195

[4] Zhou Z Ortiz Lopez HIA Pérez GE Burgos LM Farina JM Saldarriaga C. Toxoplasmosis and the heart. Curr Probl Cardiol. (2021) 46:100741. doi: 10.1016/j.cpcardiol.2020.100741 33183832

[5] Karimi P Shafaghi-Sisi S Meamar AR Nasiri G Razmjou E. Prevalence and molecular characterization of *toxoplasma gondii* and Toxocara cati among stray and household cats and cat owners in Tehran, Iran. Front Vet Sci. (2022) 9:927185. doi: 10.3389/fvets.2022.927185, PMID: 35812883 PMC9257223

[6] Liu Q Cao L Zhu XQ. Major emerging and re-emerging zoonoses in China: a matter of global health and socioeconomic development for 1.3 billion. Int J Infect Dis. (2014) 25:65–72. doi: 10.1016/j.ijid.2014.04.003, PMID: 24858904 PMC7110807

[7] Khan AH Noordin R. Serological and molecular rapid diagnostic tests for toxoplasma infection in humans and animals. Eur J Clin Microbiol Infect Dis. (2020) 39:19–30. doi: 10.1007/s10096-019-03680-2, PMID: 31428897 PMC7087738

[8] Cesbron-Delauw MF. Dense-granule organelles of *toxoplasma gondii*: their role in the host-parasite relationship. Parasitol Today. (1994) 10:293–6. doi: 10.1016/0169-4758(94)90078-7 15275422

[9] Hughes HP van Knapen F. Characterisation of a secretory antigen from *toxoplasma gondii* and its role in circulating antigen production. Int J Parasitol. (1982) 12:433–7. doi: 10.1016/0020-7519(82)90073-X 7141782

[10] Griffith MB Pearce CS Heaslip AT. Dense granule biogenesis, secretion, and function in *toxoplasma gondii*. J Eukaryot Microbiol. (2022) 69:e12904. doi: 10.1111/jeu.12904 35302693 PMC9482668

[11] Ybanez RHD Ybanez AP Nishikawa Y. Review on the current trends of toxoplasmosis Serodiagnosis in humans. Front Cell Infect Microbiol. (2020) 10:204. doi: 10.3389/fcimb.2020.00204, PMID: 32457848 PMC7227408

[12] Ybañez RH Nishikawa Y. Comparative performance of recombinant GRA6, GRA7, and GRA14 for the Serodetection of toxoplasma gondii infection and analysis of IgG subclasses in human sera from the Philippines. Pathogens. (2022) 11:277. doi: 10.3390/pathogens11020277, PMID: 35215219 PMC8874886

[13] Yang J Ai J Qi T Ni X Xu Z Guo L. *Toxoplasma gondii* and *Neospora caninum* infections in stray cats and dogs in the Qinghai-Tibetan plateau area, China. Animals (Basel). (2022) 12:1390. doi: 10.3390/ani12111390 35681854 PMC9179287

[14] Döşkaya M Liang L Jain A Can H Gülçe İz S Felgner PL. Discovery of new *toxoplasma gondii* antigenic proteins using a high throughput protein microarray approach screening sera of murine model infected orally with oocysts and tissue cysts. Parasit Vectors. (2018) 11:393. doi: 10.1186/s13071-018-2934-1, PMID: 29973272 PMC6033234

[15] Wang R Wu M Cai H An R Chen Y Wang J. Preparation and preliminary application of epitope peptide-based antibody against *toxoplasma gondii* GRA3. Trop Med Infect Dis. (2023) 8:143. doi: 10.3390/tropicalmed8030143 36977144 PMC10053247

[16] Salman D Oohashi E Mohamed AEA Abd El-Mottelib AER Okada T Igarashi M. Seroprevalences of *toxoplasma gondii* and *Neospora caninum* in pet rabbits in Japan. J Vet Med Sci. (2014) 76:855–62. doi: 10.1292/jvms.13-0632, PMID: 24584081 PMC4108769

[17] Al-Adhami BH Simard M Hernández-Ortiz A Boireau C Gajadhar AA. Development and evaluation of a modified agglutination test for diagnosis of toxoplasma infection using tachyzoites cultivated in cell culture. Food Waterborne Parasitol. (2016) 2:15–21. doi: 10.1016/j.fawpar.2015.12.001

[18] Desmonts G Remington JS. Direct agglutination test for diagnosis of toxoplasma infection: method for increasing sensitivity and specificity. J Clin Microbiol. (1980) 11:562–8. doi: 10.1128/jcm.11.6.562-568.1980, PMID: 7000807 PMC273461

[19] Hiszczyñska-Sawicka E Kur J Pietkiewicz H Holec L Gasior A Myjak P. Efficient production of the *Toxoplasma gondii* GRA6, p35 and SAG2 recombinant antigens and their applications in the serodiagnosis of toxoplasmosis. Acta Parasitol. (2005) 50:249–54.

[20] Hiszczyńska-Sawicka E Brillowska-Dąbrowska A Dąbrowski S Pietkiewicz H Myjak P Kur J. High yield expression and single-step purification of toxoplasma gondii SAG1, GRA1, and GRA7 antigens in *Escherichia coli*. Protein Expr Purif. (2003) 27:150–7. doi: 10.1016/S1046-5928(02)00593-4, PMID: 12509997

[21] Attias M Teixeira DE Benchimol M Vommaro RC Crepaldi PH De Souza W. The life-cycle of *toxoplasma gondii* reviewed using animations. Parasit Vectors. (2020) 13:588. doi: 10.1186/s13071-020-04445-z, PMID: 33228743 PMC7686686

[22] Gatkowska J Hiszczynska-Sawicka E Kur J Holec L Dlugonska H. *Toxoplasma gondii*: an evaluation of diagnostic value of recombinant antigens in a murine model. Exp Parasitol. (2006) 114:220–7. doi: 10.1016/j.exppara.2006.03.011, PMID: 16707125

[23] Holec-Gasior L. *Toxoplasma gondii* recombinant antigens as tools for serodiagnosis of human toxoplasmosis: current status of studies. Clin Vaccine Immunol. (2013) 20:1343–51. doi: 10.1128/CVI.00117-13, PMID: 23784855 PMC3889578

[24] Lecordier L Fourmaux MP Mercier C Dehecq E Masy E Cesbron-Delauw MF. Enzyme-linked immunosorbent assays using the recombinant dense granule antigens GRA6 and GRA1 of toxoplasma gondii for detection of immunoglobulin G antibodies. Clin Diagn Lab Immunol. (2000) 7:607–11. doi: 10.1128/CDLI.7.4.607-611.2000, PMID: 10882660 PMC95922

[25] Cai Y Wang Z Li J Li N Wei F Liu Q. Evaluation of an indirect ELISA using recombinant granule antigen Gra7 for serodiagnosis of *toxoplasma gondii* infection in cats. J Parasitol. (2015) 101:37–40. doi: 10.1645/14-575.1, PMID: 25216850

[26] Suwan E Chalermwong P Rucksaken R Sussadee M Kaewmongkol S Udonsom R. Development and evaluation of indirect enzyme-linked immunosorbent assay using recombinant dense granule antigen 7 protein for the detection of toxoplasma gondii infection in cats in Thailand. Vet World. (2022) 15:602–10. doi: 10.14202/vetworld.2022.602-610, PMID: 35497967 PMC9047132

[27] Ybañez RHD Kyan H Nishikawa Y. Detection of antibodies against *toxoplasma gondii* in cats using an immunochromatographic test based on GRA7 antigen. J Vet Med Sci. (2020) 82:441–5. doi: 10.1292/jvms.19-0654, PMID: 32037381 PMC7192716

[28] Jacobs D Vercammen M Saman E. Evaluation of recombinant dense granule antigen 7 (GRA7) of *toxoplasma gondii* for detection of immunoglobulin G antibodies and analysis of a major antigenic domain. Clin Diagn Lab Immunol. (1999) 6:24–9. doi: 10.1128/CDLI.6.1.24-29.1999, PMID: 9874659 PMC95655

[29] Kotresha D Poonam D Muhammad Hafiznur Y Saadatnia G Nurulhasanah O Sabariah O. Recombinant proteins from new constructs of SAG1 and GRA7 sequences and their usefulness to detect acute toxoplasmosis. Trop Biomed. (2012) 29:129–37. PMID: 22543613

[30] Lau YL Hasan MT Thiruvengadam G Idris MM Init I. Cloning and expression of toxoplasma gondii dense granular protein 4 (GRA4) in Pichia pastoris. Trop Biomed. (2010) 27:525–33. PMID: 21399595

[31] Smolskaya S Logashina YA Andreev YA. *Escherichia coli* extract-based cell-free expression system as an alternative for difficult-to-obtain protein biosynthesis. Int J Mol Sci. (2020) 21:928. doi: 10.3390/ijms21030928, PMID: 32023820 PMC7037961

[32] Calhoun KA Swartz JR. An economical method for cell-free protein synthesis using glucose and nucleoside monophosphates. Biotechnol Prog. (2005) 21:1146–53. doi: 10.1021/bp050052y, PMID: 16080695

[33] Tang W Zhang J Wang Z Hong M. The cause of deviation made in determining the molecular weight of his-tag fusion proteins by SDS-PAGE. Acta Phytophysiol Sin. (2000) 26:64–8.

