# Supplementary material for: Comparison of the performance of SAG2, GRA6, and GRA7 for serological diagnosis of Toxoplasma gondii infection in cats
Source: Front Vet Sci. 2024 Jun 5;11:1423581. doi: 10.3389/fvets.2024.1423581 (PMC11186378; doi:10.3389/fvets.2024.1423581)
Supplement: Supplementary file 1 [file Table_1.DOCX]

| **Fragments** | **Primer（5´-3´） Sequence** | **Size (bp)** |
| --- | --- | --- |
| TgGRA3 | GRA3-F: TCACTAGTTCTCGAGCTCGGTACCATGGACCGTACCATATGT  GRA3-R: TCAagcgtaatccggcacatcgtaggggtaGGTACCGGTTTGTTTCTTGGAGGC | 702 |
| TgGRA4-T | GRA4-T-F: TCACTAGTTCTCGAGCTCGGTACCATGGGTGAGTGCAGCTTTGGT  GRA4-T-R: TCAagcgtaatccggcacatcgtaggggtaCATATGCTCTTTGCGCATTCTTTC | 1011 |
| TgGRA4 | GRA4-F: TCACTAGTTCTCGAGCTCGGTACCATGCAGGGCACTTGGTTT  GRA4-R: TCAagcgtaatccggcacatcgtaggggtaCATATGCTCTTTGCGCATTCTTTC | 1071 |
| TgGRA5-T | GRA5-T-F: TCACTAGTTCTCGAGCTCGGTACCATGTTAATTTTTGTGGGCGTT  GRA5-T-R: cggcacatcgtaggggtaCATATGCTCTTCCTCGGCAACTTC | 342 |
| TgGRA5 | GRA5-F: TCACTAGTTCTCGAGCTCGGTACCATGGCGTCTGTAAAACGC  GRA5-R: cggcacatcgtaggggtaCATATGCTCTTCCTCGGCAACTTC | 396 |
| TgGRA6 | GRA6-F: TCACTAGTTCTCGAGCTCGGTACCATGGCACACGGTGGCATC  GRA6-R: cggcacatcgtaggggtaCATATGATAATCAAACACATTCAC | 705 |
| TgGRA8 | GRA8-F: TCACTAGTTCTCGAGCTCGGTACCATGGCTTTACCATTGCGT  GRA8-R: cggcacatcgtaggggtaCATATGATTCTGCGTCGTTACGGT | 837 |
| TgGRA8-T | GRA8-T-F: TCACTAGTTCTCGAGCTCGGTACCATGATGAACGGTCCTTTGAGT  GRA8-T-R: cggcacatcgtaggggtaCATATGATTCTGCGTCGTTACGGT | 768 |
| TgGRA14-T | GRA14-T-F: TCACTAGTTCTCGAGCTCGGTACCATGGCTGCCAGTTTGGAGCAG GRA14-T-R: TCAagcgtaatccggcacatcgtaggggtaCATATGTTCGCTTGGTCTCTGGTA | 1161 |
| TgROP6-T | ROP6-T-F: TCACTAGTTCTCGAGCTCGGTACCATGGTCTGCGTCTCCGGTCTG  ROP6-T-R: TCAagcgtaatccggcacatcgtaggggtaCATATGCTGGCTGCCGGAATTCGC | 1299 |
| TgSRS29A-T | SRS29A-T-F: TCACTAGTTCTCGAGCTCGGTACCATGGTGAGGACATCCCTT  SRS29A-T-R: TCAagcgtaatccggcacatcgtaggggtaCATATGGACGGCACCAAACATAGC | 1260 |

**Table S1** [Primers for target genes to construct plasmids used for cell-free expression.](#_Toc69196657)
